# The Chemical Characterization of *Eleutherococcus senticosus* and Ci-wu-jia Tea Using UHPLC-UV-QTOF/MS

**DOI:** 10.3390/ijms20030475

**Published:** 2019-01-22

**Authors:** Yan-Hong Wang, Yonghai Meng, Chunmei Zhai, Mei Wang, Bharathi Avula, Jimmy Yuk, Kerri M. Smith, Giorgis Isaac, Ikhlas A. Khan

**Affiliations:** 1 National Center for Natural Products Research, Research Institute of Pharmaceutical Sciences, School of Pharmacy, University of Mississippi, University, MS 38677, USA; wangyh@olemiss.edu (Y.-H.W.); meiwang@olemiss.edu (M.W.); bavula@olemiss.edu (B.A.); 2 School of Pharmacy, Heilongjinag University of Chinese Medicine, Harbin 150040, China; myhdx163@163.com (Y.M.); zhaicm163@163.com (C.Z.); 3 Waters Corporation, Milford, MA 01757, USA; Jimmy_Yuk@waters.com (J.Y.); Kerri_Smith@waters.com (K.M.S.); Giorgis_Isaac@waters.com (G.I.); 4 Division of Pharmacognosy, Department of BioMolecular Sciences, School of Pharmacy, University of Mississippi, MS 38677, USA

**Keywords:** *Eleutherococcus senticosus*, *Acanthopanax senticoccus*, triterpene glycosides, flavonoid, quinic acid, UNIFI

## Abstract

*Eleutherococcus senticosus* Maxim. belongs to the Araliaceae family. Phytochemical studies reveal that *E. senticosus* leaves contain triterpene glycosides along with organic acid derivatives and flavonoid compounds. It is believed that *E. senticosus* is similar to ginseng because they come from same family and both contain triterpene saponins. *E. senticosus* leaves have been developed as a functional beverage called ci-wu-jia tea in recent years. Triterpene glycosides are difficult to identify by ultraviolet (UV) detection and contents of these compounds are low in *E. senticosus* leaves. In this study, a sensitive ultra-high performance liquid chromatographic (UHPLC) method combining UV and tandem mass spectrometry (MS/MS) was developed to characterize the triterpene glycosides from *E. senticosus* leaves and related commercial products. Fragmentation patterns of three sub-groups of triterpene glycosides in *E. senticosus* leaves were investigated. Additionally, fragmentation pathways and UV characteristics of organic acid derivatives and flavonoids were also characterized. A compound screening library, including 241 compounds reported in the literature, was created and used to confirm the compounds in the samples. In this study, a total of 24 samples, including 13 plant samples of *E. senticosus* and 11 ci-wu-jia tea products, were analyzed. Out of the 11 commercial products, three products were discovered to contain green tea (*Camellia sinensis)* that was considered to be an adulterant since it was not an ingredient on the labels. The developed UHPLC-UV-MS/MS analytical method combined with the UNIFI processing method can simultaneously characterize organic acid derivatives, flavonoids, and triterpene saponins from *E. senticosus*. It provides a simple and sensitive way to perform quality control of *E. senticosus* and related ci-wu-jia tea products.

## 1. Introduction

*Eleutherococcus senticosus* Maxim. (syn. *Acanthopanax senticosus* Harms) is a species of the Araliaceae family. It usually grows in forests or thickets, where it is elevated from hundreds to above 2000 m in altitude in China. Globally, this plant is distributed in Russia and East Asia, including China, Korea, and Japan. *E. senticosus* is also known as ci-wu-jia in China and mainly grows in Shanxi, Hebei, and the north-eastern region of China [1]. Roots, rhizomes, or stems of *E. senticosus* are collected in the spring or fall and used as a tonic and anti-fatigue agent to invigorate *qi*, strengthen the spleen, and nourish the kidney in the theory of traditional Chinese medicine (TCM) [2]. *E. senticosus* is in the same family as *Panax ginseng* [3] and the leaves have been reported for glycosidase inhibition as well as having antibacterial properties [4]. For this reason, *E. senticosus* leaves have been developed as a functional beverage called ci-wu-jia tea in China and Siberian ginseng tea in the United States and Europe. Phytochemical studies revealed that caffeoylquinic acid derivatives, triterpene glycosides, and flavonoids were the major secondary metabolites in leaves of *E. senticosus* [3,5].

Several analytical methods have been reported for the quantitative or qualitative analysis of caffeoylquinic acid derivatives, triterpene glycosides, and flavonoids in *E. senticosus* leaves [3,6,7,8,9]. In earlier studies, Liu’s research team summarized the fragmentation patterns of flavonoids and triterpene glycosides by tandem mass spectrometry [6,7]. The same team also reported a group of caffeoylquinic acid derivatives and flavonoids in extracts of *E. senticosus* leaves by high performance liquid chromatography coupled with diode array detector and tandem mass spectrometer (HPLC-DAD-MS/MS). These identified compounds showed potential α-glucosidase inhibition [8]. In recent years, Xiaodan Zhang et al. quantified rutin, quercetin-3-*O*-glucoside, kaempferol-3-*O*-rutinoside, and six caffeoylquinic acid derivatives from different species of *Acanthopanax* [3]. Yue-Wei Ge et al. developed an MS/MS similarity network-based approach for chemical profiling of saponins in *E. senticosus* leaves [9]. However, no analytical method has been reported for the chemical analysis of caffeoylquinic acid derivatives, flavonoids, and triterpene glycosides in a single analytical method. Due to the higher sales of commercial natural products in the market, there have been increasing cases of intentional adulteration with different species to lower the cost [10]. To ensure quality control of ci-wu-jia or Siberian ginseng tea in the market, it is important to develop an analytical method for the characterization of these types of major constituents in *E. senticosus* leaves. In this study, an analytical method was developed to characterize the different classes of compounds (organic acid derivatives, flavonoids, and triterpene glycosides) in *E. senticosus* leaves using a single ultra-high performance liquid chromatography (UHPLC) method coupled to photo-diode array (PDA) and quadrupole time-of-flight mass Spectrometer (QToF MS) detectors. Compounds’ characterization was carried out by combining UV, MS, and MS/MS data with the informatics platform, UNIFI. Characteristic UV, MS, and MS/MS spectra of typical compounds in each class were defined. The commonly observed fragment ions and neutral loss were determined and edited into the UNIFI processing method. The UHPLC-UV-MS/MS data of authentic plant samples were processed and marker compounds were confirmed by comparison with the retention time, UV and MS spectra, as well as the MS/MS fragmentation pattern of reference standards and the literature. The analyzed samples or products not fully matched with the authentic material were identified. Compounds causing the differences in those non-matching samples/products were determined and characterized using the established personal library. In total, 13 plant samples collected from different locations and 11 ci-wu-jia teas were tested using the developed method. Out of the 11 ci-wu-jia tea samples, three (EPS-1, EPS-2, and EPS-8) products were determined to contain caffeine, epigallocatechin, epicatechin, epigallocatechin gallate, and epicatechin gallate; all compounds that matched with the profile of green tea extract. These results indicate that the products, EPS-1, EPS-2, and EPS-8, contained green tea. However, green tea was a non-listed ingredient on the products’ label and is considered to be an adulterant in these Ci-wu-jia tea products. To the author’s knowledge, this is the first comprehensive study for the development of a single analytical method for the chemical compounds in *E. senticosus* leaves using UHPLC-UV-QTOF/MS with the analysis of commercial products in the market.

## 2. Results and Discussion

The chemical constituents of *E. senticosus* leaves include hydrophilic compounds, such as organic acid derivatives, flavonoids, as well as triterpene glycosides. It can be a challenge to retain and separate these compound classes on most of C18 reversed phase columns. The high strength silica (HSS) column enables polar compounds to more readily access the pore structure of the solid material and increases their retention time. In this work, the developed UHPLC-UV-MS/MS method is optimized with UHPLC columns, column temperature, mobile phase, gradient elution method, flow rate, and MS responses on a UHPLC system coupled to a quadrupole time-of-flight mass spectrometer with electrospray ionization. As a result, acetonitrile-water with 0.05% formic acid combined with the optimized gradient elution on an UPLC HSS T3 column (1.8 µm, 2.1 × 100 mm i.d.) afforded the best separation and MS response in the positive mode to simultaneously identify different classes of compounds, such as organic acid derivatives, flavonoids, and triterpene glycosides, in a single injection analysis. This is the first method to focus on full characterization of the main components in *E. senticosus* leaves.

**Figure 1:**
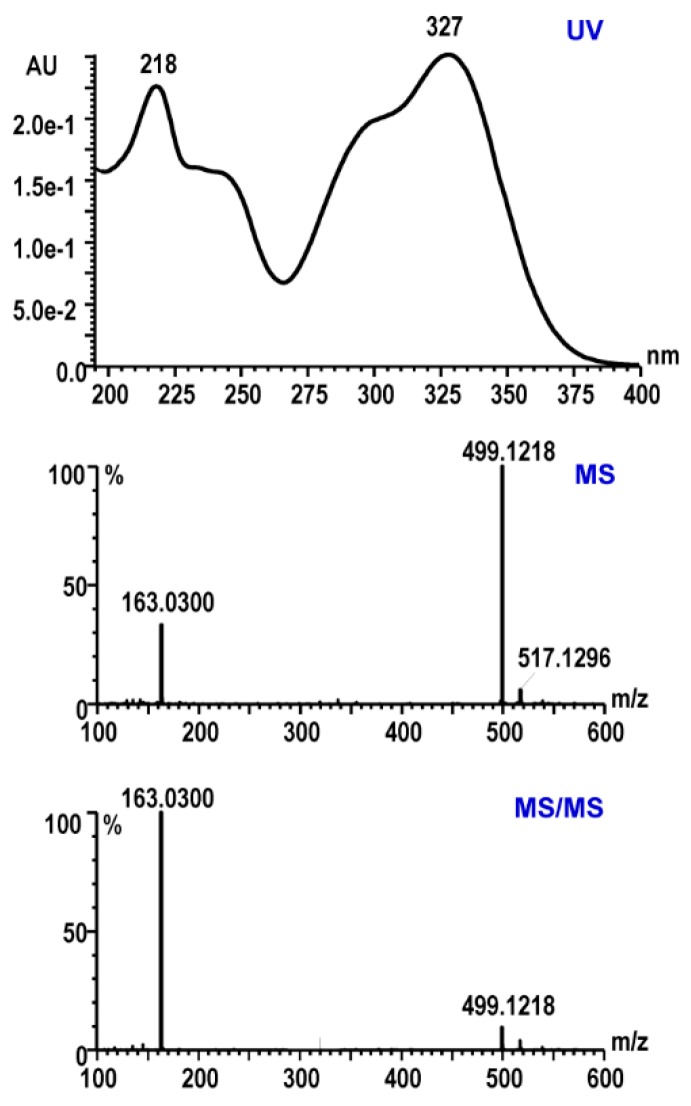
UV, MS, and MS/MS spectra of 3,5-dicaffeoylquinic acid.

Organic acid derivatives, such as chlorogenic acid and 3,5-dicaffeoylquinic acid, have been reported from *E. senticosus*. As the phenolic group conjugates with the α,β-unsaturated acid unit in caffeic acid or ferulic acid molecules, the organic acid derivatives have characteristic UV absorption around 218, 243, 295 (shoulder), and 327 nm. A peak of λ_max_ at 327 nm with a shoulder at 295 nm is a unique signature of organic acid derivatives and can be used to identify this family from other classes of compounds in *E. senticosus*. In addition, MS and MS/MS spectra of organic acid derivatives contain common fragment ions at *m/z* 163 or 177 Da corresponding to the loss of caffeic acid or ferulic acid, respectively. In *E. senticosus* leaves, 3,5-dicaffeoylquinic acid elutes at 8.72 min with the typical UV absorption of organic acid derivatives and showed pronated molecular ions at *m/z* 517.1343 Da ([C_25_H_25_O_12_]^+^, *calc.* 517.1346) (Figure 1). The MS/MS spectrum of 3,5-dicaffeoylquinic acid, fragment ions at *m/z* 499.1218, 355.0952, 337.0863, and 163.0300 Da, respectively, correspond to [M-H_2_O]^+^, [M-caffeoyl unit]^+^, [M-H_2_O-caffeoyl unit]^+^, and [Caffeoyl unit]^+^. 3,5-Dicaffeoylquinic acid is unambiguously identified by comparing the retention time, UV, MS, and MS/MS spectra with that of the reference standard. In total, 13 organic acid derivatives are characterized from the leaves of *E. senticosus* (Table 1).

Flavonoids are another group of compounds in *E. senticosus* leaves with typical UV absorptions around 203, 255, and 354 nm that are related to flavonoid core quercetin or kaempferol. These compounds contain key fragment ions at *m/z* 303 or 287 Da corresponding to aglycone quercetin or kaempferol, respectively, in the MS and MS/MS spectra. Further, a neutral loss of 162 or 146 Da related to the loss of hexose and deoxyhexose, respectively, are commonly found for flavonoid glycosides. In *E. senticosus* leaves, rutin eluted at 7.22 min and is confirmed by comparing the retention time, UV, MS, and MS/MS spectra with that of a standard compound. Key UV absorption and MS/MS fragments of rutin are listed in Table 1 along with the other six flavonoid glycosides.

**Figure 2:**
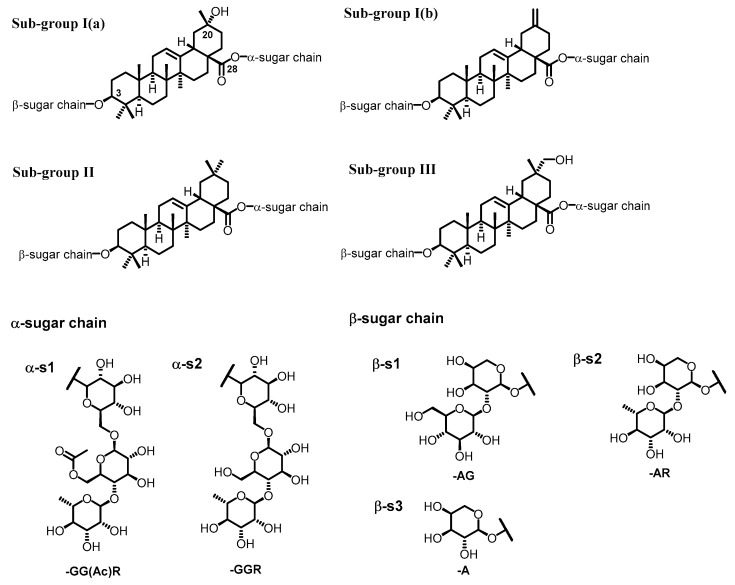
Aglycones of three sub-groups of triterpene glycosides in *E. senticosus.*

Triterpene glycoside compounds are key constituents in *E. senticosus* leaves, but show weak UV absorption due to a lack of obvious chromophore on the triterpene aglycone. Investigation of the MS/MS fragmentation pattern provides a tool to characterize this class of compounds. Typical sub-groups of triterpene glycosides were classified according to the difference of substitution or oxidization at C-20 (Figure 2). Oleanolic acid and akebonoic acid are two classical core skeletons of triterpene glycosides found in *E. senticosus* leaves [3,8]. As shown in Figure 2, α- and β-sugar chains connect at C-28 and C-3, respectively. The sugar chains, α-S1 and α-S2, both contain the moiety glucose-glucose-rhamnose, but α-S1 has an extra acetyl group connecting at C-6 of the second glucose [3]. MS/MS spectra of α-S1 and α-S2 show fragments of *m/z* 513 and 471, respectively, corresponding to dissociation of α-S1 or α-S2 from C-28. Following that, α-S1 produces fragments at *m/z* 367 and 205, respectively, corresponding to ions of [513-rhamnose]^+^ and [glucose + acetyl group − H_2_O]^+^; α-S2 yields fragments at *m/z* 325 and 147 corresponding to ions of [471-rhamnose]^+^ and [rhamnose + H – H_2_O]^+^, respectively. For β-sugar chains, sugars usually dissociated one-by-one from the very end of the chain. The fragments, [M-162]^+^, [M-146]^+^, and [M-132]^+^, correspond to the loss of glucose, rhamnose, and arabinose, respectively, from the end of β-sugar chains. MS and MS/MS key fragments of *m/z* 441/423 pair, 423, 439, and 455 Da are very important keys to determine the saponins in sub-group I(a), I(b), II, and III, respectively (Figure 2).

**Figure 3:**
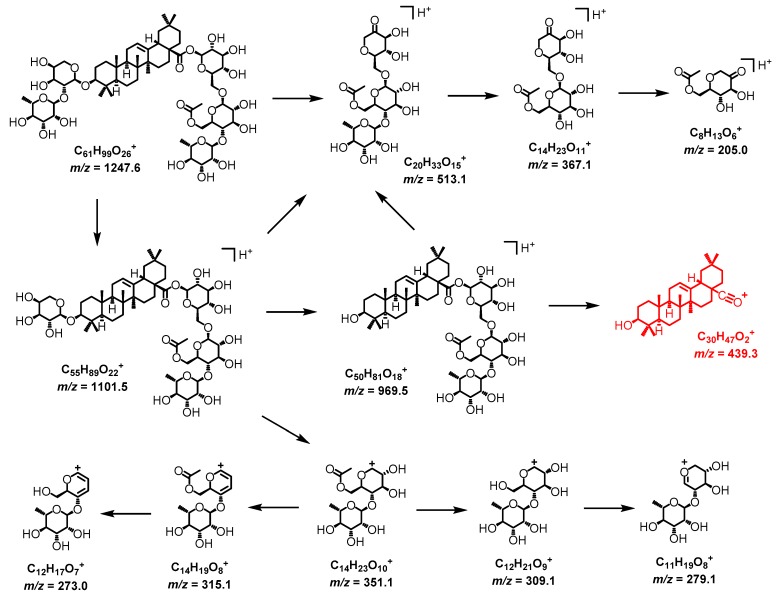
Proposed mass fragmentation pathway for ciwujianoside C4 (key fragment of sub-group II at *m/z* = 439.3 Da is shown in red color).

Ciwujianoside C4 eluted at 17.31 min. For the MS spectrum of ciwujianoside C4 (Figure S1), pronated molecular ions and ammonium adduct ions are found at *m/z* 1247.6813 ([C_61_H_99_O_26_]^+^, *calc.* 1247.6419) and 1264.7212 Da ([C_61_H_102_NO_26_]^+^, *calc.* 1264.6685), respectively. For the MS/MS spectrum (Figure S1), a key fragment at *m/z* 439.3 Da indicates that ciwujianoside C4 contains the aglycone of sub-group II saponins. In addition, fragments at *m/z* 513.1, 367.1, and 205.0 Da correspond to the loss of sugars of the α-S1 sugar chain at C-28 (Figure 3); fragments at *m/z* 1101.6 and 969.5 Da are related to the loss of rhamnose and arabinose from C-3, respectively. Therefore, the fragmentation pathway of ciwujianoside C4 is proposed in Figure 3 in which ions of *m/z* 351.1, 315.1, 309.1, 279.1, and 273.0 Da are essential fragments related to the α-S1 sugar chain.

Saponins in sub-group I(a), I(b), and III present very similar fragmentation patterns as sub-group II triterpene glycosides, such as ciwujinoside C4. Determination of sugar chains at C-3 and C-28 can follow the same rule as the ciwujianoside C4 example in Figure 3. The key point is to identify the core skeleton of saponins’ aglycone. MS and MS/MS spectra of sub-group I(a) usually shows ion pairs at *m/z* 441.3 and 423.3 Da, but sub-group I(b) only shows ions at *m/z* 423.3 Da. In sub-group III saponins, ions at *m/z* 455.3 Da are fragments indicating the existence of the sub-group III core (Figure 2). In summary, the characterization of key fragments of 441/423 pair, 423, 439, and 455 Da can differentiate compounds in sub-group I(a), I(b), II, and III. Sugar chains at C-28 (α-S1 and α-S2) easily break down completely and the newly forming α-sugar chains fragments are more likely to yield sugar fragments step by step as shown in Figure 3. The sugar chain at C-3 (β-sugar chains, Figure 2) usually dissociates one-by-one from the far end of sugar chains. Therefore, C-3 and C-28 can be determined on the basis of MS and MS/MS along with reference standards and known values from the literature. In this work, a total of 30 saponins are characterized in Table 1.

To create a processing method for the quality control analysis of *E. senticosus* and related products, MS and MS/MS raw data of authentic plant samples, reference standards, and testing samples are imported into an LC-UV-MS, the screening platform, UNIFI, for characterization. A personal library with 241 entries of compounds identified from *Eleutherococus* species and green tea is compiled and used for the screening of 13 of *E. senticosus* plant material and 11 of ci-wu-jia tea products. To ensure the processing method contained all the specific parameters required to screen *E. senticosus* and related products, several iterations of method development were performed. In the preliminary UNIFI processing method, non-pecific parameters, such as peak processing settings using 3D peak apex, target by mass of targeted screen settings, common fragment and neutral loss parameters of discovery settings, and adducts and lock mass of analysis specific settings, were set up. When reviewing the processed data, the reference standards for *E. senticosus* were confirmed by screening these compounds in the authentic *E. senticosus* leaves. Information, such as the accurate mass, MS/MS fragments, UV absorption, fragmentation pattern, and retention time of the reference standards, were determined experimentally as well as referencing previous literature findings in authentic *E. senticosus* leaves [3,4,5]. Following preliminary processing, the method was updated using specific parameters. Common fragments were set up for organic acid derivatives at *m/z* 163 and 177 Da; for flavonoids at *m/z* 287 and 303 Da; and for triterpene glycosides at *m/z* 205, 273, 279, 309, 315, 351, 367, 423, 439, 441, 455, 471, and 513. A common neutral loss of 44, 132, 146, and 162 Da were included for searching for fragment ions related to CO_2_, pentose, deoxy hexose, and hexose, respectively. Using the updated method, all samples, including *E. senticosus* plant samples and commercial products, were processed again.

**Figure 4:**
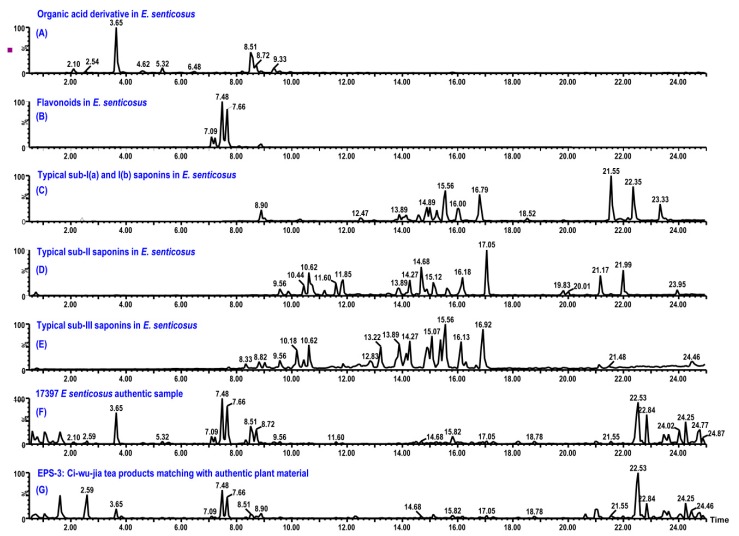
Chromatograms of organic acid derivatives (**A**), flavonoids (**B**), sub-I(a) and I(b) saponins (**C**), sub-II saponins (**D**), sub-III saponins (**E**), #17397 *E. senticosus* authentic plant sample (**F**), and typical Ci-wu-jia tea product (EPS-3) matching with plant material (**G**).

**Figure 5:**
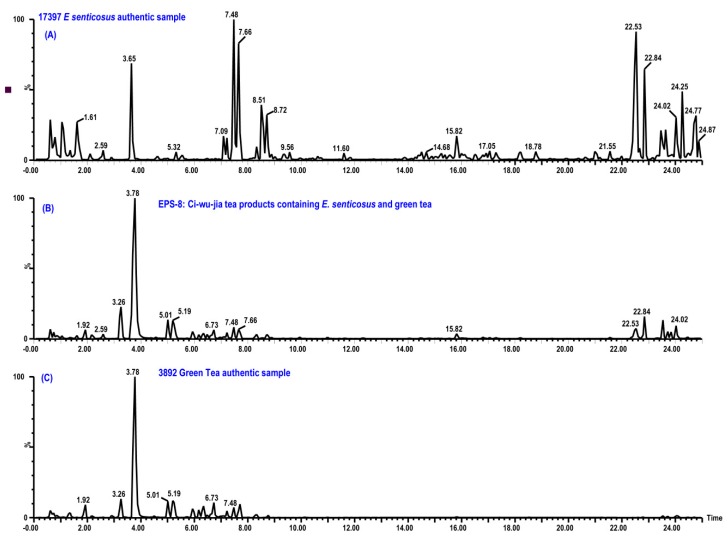
Chromatograms of #17397 *E. senticosus* authentic plant sample (**A**), typical Ci-wu-jia tea product (EPS-8) containing *E. senticosus* and green tea (**B**), and #3892 green tea authentic plant sample (**C**).

Reviewing results of all the samples, tea products of *E. senticosus* EPS-3 to EPS-7 and EPS-9 to EPS-11 have identical profiles with that of authentic *E. senticosus* leaves. Their confirmed plots and tables are same as samples of *E. senticosus* leaves (Figure 4). However, profiles of samples EPS-1, EPS-2, and EPS-8 are different with that of authentic plant material (Figure 5). Major components between 2 and 8 min are noteworthy in these *E. senticosus* tea products. The compound at 3.78 min displays a UV absorption of λ_max_ at 205 and 272 nm and molecular ions of *m/z* 195.0873 ([C_8_H_11_N_4_O_2_]^+^, *calc.* 195.0882). In addition, compounds at 3.26, 5.01, 5.19, and 7.48 min contain common key fragments of *m/z* 139 Da and similar UV absorption around 205, 230 (shoulder), and 274 nm. Using the developed personal library, which contains compounds identified from *Eleutherococus* species and green tea as well as the built-in UNIFI TCM library, the compound at 3.78 min is identified as caffeine. The components at 3.26, 5.01, 5.19, and 7.48 min were determined to be epigallocatechin, epicatechin, epigallocatechin gallate, and epicatechin gallate, respectively, by using the in-house created UNIFI library and confirmed using injections of reference standards. An authentic green tea sample is prepared and tested by the developed UHPLC-UV-MS/MS method. Products EPS-1, EPS-2, and EPS-8 are identified as green tea products mixed with *E. senticosus* leaves. However, labels of products EPS-1, EPS-2, and EPS-8 do not list green tea as an ingredient. Therefore, the green tea in products EPS-1, EPS-2, and EPS-8 is considered an adulterant that would enhance the tasting flavor and reduce the products’ cost.

## 3. Materials and Methods

### 3.1. Instrumentation and Chromatographic Conditions for UHPLC-UV-MS Analysis

All samples were analyzed by using a Waters Acquity UPLC HSS T3 column (100 × 2.1 mm i.d., 1.8 μm) on a Waters Acquity UPLC system (Waters, Milford, MA, USA) that included a binary solvent manager, sample manager, heated column compartment, photodiode array (PDA) detector, and Xevo G2-S QToF mass spectrometer. The instrument was controlled by MassLynx NT 4.1 (Waters, Milford, MA, USA). The column and sample temperature were maintained at 30 and 10 °C, respectively. The eluent consisted of water containing 0.05% formic acid (A) and acetonitrile with 0.05% formic acid (B). Analysis was performed using gradient elution at a flow rate of 0.35 mL/min as follows: 0–8 min, 8% to 23% B; 8–20 min, 23% to 45% B; 20–25 min, 45% to 85% B; 25–27 min, 85% to 100% B. The analysis was followed by a 3 min washing procedure with 100% B and re-equilibration period of 3.5 min with initial conditions. A strong needle wash solution (90/10; acetonitrile/water, *v*/*v*) and weak needle wash solution (10/90; acetonitrile/water) were used. The injection volume was 2 µL.

The high-resolution ESI-MS experiments were carried out on a Xevo G2-S QToF mass spectrometer that was connected to the UHPLC system via an ESI interface. The ESI source was operated in the positive ionization mode with the following settings of the parameters: 3.5 kV capillary voltage; 35 V cone voltage; 85 and 450 °C for ion source and desolvation temperature, respectively; and 50 and 900 L/h for cone and desolvation gas flows, respectively. Mass accuracy of the parent and major fragments in this study was limited within 5 ppm, but a few minor fragment ions were tolerated up to 10 ppm when considering its limited peak intensity in the analysis. Leucine-enkephalin was used for the lock mass at a concentration of 2 ng/mL and flow rate of 5 μL/min. Ions [M + H]^+^ (*m/z* 556.2771 Da) and a fragment ion (*m/z* 278.1141 Da) of leucine-enkephalin were employed to ensure mass accuracy during the MS analysis. The lock spray interval was set at 30 s, and the data was averaged over three scans. The mass spectrometer was programmed to step between low (10 eV) and elevated (15–35 eV) collision energies on the gas cell, using a scan time of 0.1 s per function over a mass range of *m/z* 100–1500 Da.

### 3.2. Chemicals and Reagents

Methanol, acetonitrile, and formic acid were HPLC grade and purchased from Fisher Scientific. Water for the HPLC mobile phase was purified using a Millipore Synergy UV Water Purification System (Millipore SAS, Molsheim, France).

### 3.3. Plant Material and Confiscated Products

*E. senticosus* plant samples were collected in China by Dr. Yonghai Meng. *E. senticosus* tea products were purchased from local stores in Heilongjiang, China. The information of these samples is listed in Table 2. Specimens of all samples are deposited at the Repository of Botanicals, National Center for Natural Products Research, University of Mississippi, University, Mississippi, USA.

### 3.4. Sample Preparation

Extraction method was optimized in preliminary studies to ensure that the recoveries of major components were above 95%. The fine powder of the plant material or tea product (1 g) was accurately weighed and added into a 15 mL centrifuge tube. The sample was extracted with 2.5 mL of methanol in an ultrasonic water bath for 30 min, then followed by centrifugation at 959× *g* for 15 min. The supernatant was transferred to a 10 mL volumetric flask. The procedure was repeated three more times and the respective supernatants were combined. The final volume was adjusted to 10 mL with methanol. Prior to LC analysis, the prepared sample was mixed thoroughly. An adequate volume of extract was passed through a 0.45 µm polytetrafluoroethylene (PTFE) filter and collected in an LC sample vial.

### 3.5. Data Process and Analysis

All LC-UV-MS data was processed, peak picked, and analyzed using the UNIFI informatics platform (Waters, Milford, MA, USA). A three-dimensional (3D) peak detection algorithm was used to detect the peak apexes of all the ion responses based on their 3D shapes to obtain cleaner spectra and more accurate peak volumes than 2D extracted ion chromatograms. For determination of the 3D peak apex, the retention time and intensity threshold of high and low energy were set as 0.5–25 min, 50 counts, and 100 counts, respectively. Ammonium ion (NH_4_^+^) was add as an adduct cluster for the mass defect search. Common fragments included key fragments of organic acid derivatives (*m/z* 163 and 177), flavonoids (*m/z* 287 and 303), and triterpene glycosides (*m/z* 205, 273, 279, 309, 315, 351, 367, 423, 439, 441, 455, 471, and 513). A common neutral loss mass of 44, 132, 146, and 162 were used to search for the loss of CO_2_, pentose, deoxy hexose, and hexose. Sodium and potassium adducts were considered in the analysis of triterpene glycoside. The lock mass was *m/z* 556.2771, corresponding to the molecular ion of leucine encephalin.

## 4. Conclusions

An UHPLC-UV-MS/MS method was developed for the characterization of different classes of compounds, including organic acid derivatives, flavonoids, and triterpene glycosides, in *E. senticosus* leaves and related tea products. According to the characteristic UV spectra, accurate mass, and MS/MS fragmentation mechanisms, 13 of the organic acid derivatives, seven of the flavonoids, and 30 of the triterpene glycosides were identified from *E. senticosus* leaves. A personal library of 241 entries related to the *Eleutherococcus* genus and green tea extracts was created in the UNIFI informatics platform. Using the UNIFI processing method that was established on the basis of the characteristics of identified compounds in *E. senticosus* leaves and green tea extract, 13 of *E. senticosus* leaves and 11 *Eleutherococcus* tea products were analyzed. Out of 11 commercial products, three samples, EPS-1, EPS-2, and EPS-8, were found to be adulterated with green tea. The approach in this work, which determined the UV, MS, and MS/MS characteristics of different classes of compounds in authentic samples and established a specific processing method to process and quantify testing samples, provides a comprehensive, but effective way to routinely analyze the quality control of complex products, such as herbal medicines and dietary supplements.

## Figures and Tables

**Table 1:** Organic acid derivatives, flavonoids, and triterpene saponins identified from authentic *E. senticosus* leaves.

Compound Class or Sug-Group	RT (min)	UV (nm)	MW	Key Fragments (Da)	Aglycone Fragments	α-Chain	β-Chain	Compound Name
			[M+H]^+^/ [M+NH_4_]^+^					
organic acid derivatives	2.1	216, 296, 327	355	355, 163				1-caffeoylquinic acid
	2.54		355	355, 163				5-caffeoylquinic acid
	3.65	217, 242, 300, 326	355	355, 163				3-caffeoylquinic acid
	3.87	215, 296, 326	355	355, 163				4-caffeoylquinic acid
	5.24	216, 241, 2986, 326	517	517, 499, 163				1,3-dicaffeoylquinic acid
	5.55	212, 296, 325	369	369, 177				3-feruloylquinic acid
	5.93		517	517, 499, 163				3,4-dicaffeoylquinic acid
	8.2	218, 298, 326	517	517, 499, 163				1,4-dicaffeoylquinic acid
	8.51	218, 243, 298, 328	517	517, 499, 355, 337, 163				1,5-dicaffeoylquinic acid
	8.72	218, 243, 295, 327	517	517, 499, 355, 337, 163				3,5-dicaffeoylquinic acid
	9.33	218, 242, 298, 327	517	517, 499, 355, 337, 163				4,5-dicaffeoylquinic acid
	10.31	218, 296, 328	531	531, 513, 177				1-caffeoyl-3-feruloylquinic acid
	10.44	218, 296, 328	531	531, 513, 177				3-feruloyl-5-caffeoylquinic acid
flavonoids	7.09	207, 255, 352	611	611, 465, 303				quercetin-3-*O*-(6-*O*-β-l-rhammanopyranose)-β-d-galactopyranose
	7.22	208, 254, 352	611	611, 465, 303				rutin
	7.48	204, 255, 354	465	465, 303				hyperoside
	7.66	204, 255, 351	465	465, 303				isoquercitrin
	8.33	217, 264, 332	449	449, 287				kampferol 3-galactoside
	8.77		449	449, 287				astragalin
	8.9		303	303				quercitrin
Saponins sub-group I(a)	9.28		1265, 1282	1103, 971, 953, 807, 603, 513, 441, 423, 367, 309, 205	441/423	GG(Ac)R	AG	GA-I(a)-GG(Ac)R
	10.25		1103, 1120	971, 953, 807, 603, 513, 441, 423, 367, 205,	441/423	GG(Ac)R	A	A-I(a)-GG(Ac)R
Saponins sub-group I(b)	14.81		1189, 1206	1027, 895, 749, 471, 423, 325, 309, 147	423	GGR	AR	RA-I(b)-GGR
	15.22		1247, 1264	1085, 953, 513, 423, 367, 309, 205	423	GG(Ac)R	AG	GA-I(b)-GG(Ac)R
	15.53		1043, 1060	911, 603, 471, 423, 325, 147	423	GGR	A	A-I(b)-GGR
	16.02		1231, 1248	1085, 953, 791, 513, 423, 367, 293, 205	423	GG(Ac)R	AR	RA-I(b)-GG(Ac)R
	16.82		1085, 1102	953, 791, 513, 423, 367, 205	423	GG(Ac)R	A	A-I(b)-GG(Ac)R
	21.53		735, 752	555, 423	423		AG	GA-I(b)
	22.38		719, 736	555, 423	423		AR	RA-I(b)
Saponins sub-group II	15.35		1221, 1238	1059, 927, 471, 439, 325, 309, 147	439	GGR	AG	GA-II-GGR
	16.48		1263, 1280	1101, 969, 513, 439, 367, 205	439	GG(Ac)R	AG	GA-II-GG(Ac)R
	16.9		1059, 1076	927, 471, 439, 325, 147	439	GGR	A	A-II-GGR
	17.31		1247, 1264	1101, 969, 513, 439, 367, 351, 315, 309, 279, 205	439	GG(Ac)R	AR	RA-II-GG(Ac)R
	18.18		1101, 1118	969, 513, 439, 367, 351, 315, 309, 279, 205	439	GG(Ac)R	A	A-II-GG(Ac)R
	22.64		751, 768	589, 439	439		AG	GA-II
	23.38		735, 752	589, 439	439		AR	RA-II
	24.41		589, 606	439	439		A	A-II
Saponins sub-group III	9.59		1237, 1254	1075, 943, 471, 455, 325, 309, 147	455	GGR	AG	GA-III-GGR
	10.41		1221, 1238	1075, 943, 617, 471, 455, 325, 293, 147	455	GGR	AR	RA-III-GGR
	10.64		1075, 1092	943, 617, 471, 455, 325, 309, 147	455	GGR	A	A-III-GGR
	10.72		1279, 1296	1117, 985, 513, 455, 367, 309, 205	455	GG(Ac)R	AG	GA-III-GG(Ac)R
	11.57		1263, 1280	1117, 985, 513, 455, 367, 293, 205	455	GG(Ac)R	AR	RA-III-GG(Ac)R
	11.83		1117, 1134	985, 513, 455, 367, 205	455	GG(Ac)R	A	A-III-GG(Ac)R
	13.86		1221, 1238	1075, 943, 471, 455, 325, 293, 147	455	GGR	AR	RA-III-GGR
	14.22		1075, 1092	943, 471, 455, 325, 293, 147	455	GGR	A	A-III-GGR
	14.74		767, 784	605, 455	455		AG	GA-III
	15.15		1263, 1280	1117, 985, 513, 455, 367, 293, 205	455	GG(Ac)R	AR	RA-III-GG(Ac)R
	15.66		1117, 1134	985, 513, 455, 367, 205	455	GG(Ac)R	A	A-III-GG(Ac)R
	17.07		605, 622	455	455		A	A-III
	19.78		767, 784	587, 455	455	G	A	A-III-G

**Table 2:** *Eleutherococcus senticosus* plant samples and Ci-wu-jia tea products used in this study.

Sample Type	Sample ID	Sample/Product Name	Plant Part
Plant Material	EPM-1 (NCNPR #17397)	*Eleutherococcus senticosus*	Leaf
	EPM-2	*Eleutherococcus senticosus*	Leaf
	EPM-3	*Eleutherococcus senticosus*	Leaf
	EPM-4	*Eleutherococcus senticosus*	Leaf
	EPM-5	*Eleutherococcus senticosus*	Leaf
	EPM-6	*Eleutherococcus senticosus*	Leaf
	EPM-7	*Eleutherococcus senticosus*	Leaf
	EPM-8	*Eleutherococcus senticosus*	Leaf
	EPM-9	*Eleutherococcus senticosus*	Leaf
	EPM-10	*Eleutherococcus senticosus*	Leaf
	EPM-11	*Eleutherococcus senticosus*	Leaf
	EPM-12	*Eleutherococcus senticosus*	Leaf
	EPM-13	*Eleutherococcus senticosus*	Leaf
Ci-wu-jia Tea Product	EPS-1	Ci-wu-jia, Heilongjiang Techan	Leaf
	EPS-2	Ci-wu-jia Teji Cha, Dongbei Techan	Leaf
	EPS-3	Ci-wu-jia Chun Tianran, Jiankang Cha	Leaf
	EPS-4	Ci-wu-jia Cha, Tianran Yesheng, Dongbei Techan	Leaf
	EPS-5	Ci-wu-jia, Heilongjiang Techan	Leaf
	EPS-6	Yesheng Ci-wu-jia Cha	Leaf
	EPS-7	Ci-wu-jia, Teji Cha Zhongguo, Heilong Jiang	Leaf
	EPS-8	Ci-wu-jia Cha	Leaf
	EPS-9	Ci-wu-jia Cha	Leaf powder
	EPS-10	Ci-wu-jia	Leaf powder
	EPS-11	Ci-wu-jia	Leaf powder

## Supplementary Materials

Supplementary materials can be found at http://www.mdpi.com/1422-0067/20/3/475/s1.

## Abbreviations

UHPLC	Ultra-high performance liquid chromatography
PDA	Photo-diode array
QToF	Quadrupole time-of-flight
MS	Mass spectrometer
TCM	Traditional Chinese medicine
