# Supplementary figures and images for: The Chemical Characterization of Eleutherococcus senticosus and Ci-wu-jia Tea Using UHPLC-UV-QTOF/MS
Source: Int J Mol Sci. 2019 Jan 22;20(3):475. doi: 10.3390/ijms20030475 (PMC6387334; doi:10.3390/ijms20030475)

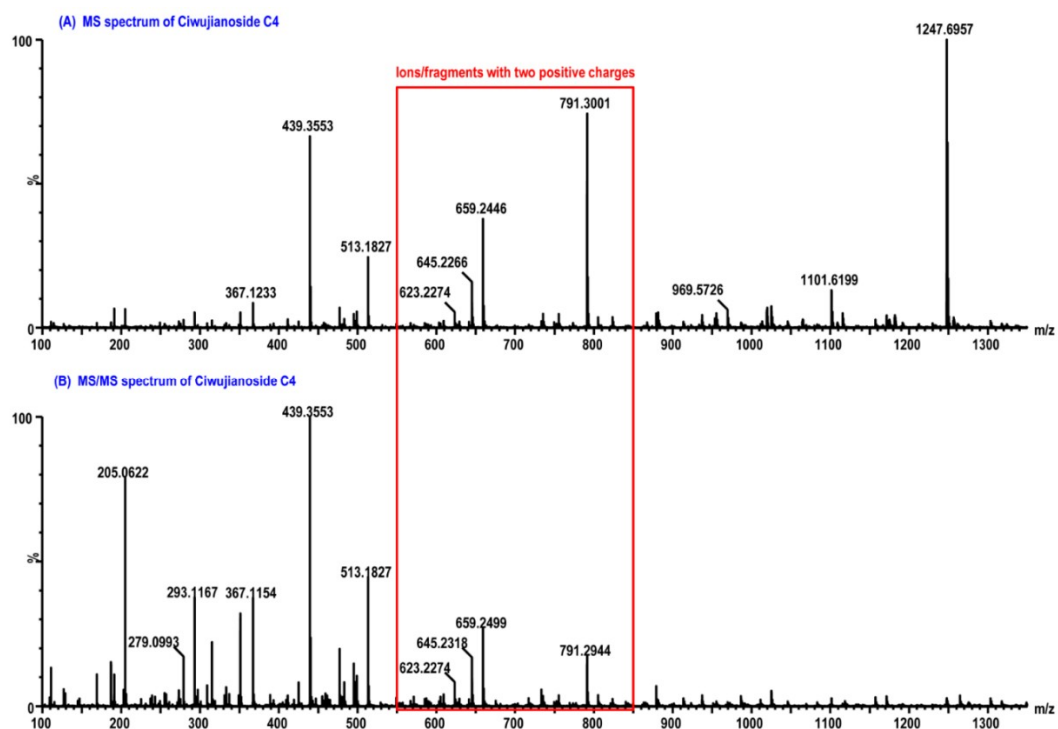

Figure S1. MS (A) and MS/MS (B) spectra of ciwujianoside C4.

Supplement: Supplementary file 1 [file ijms-20-00475-s001.pdf]
